# Highly elevated serum lactate dehydrogenase is associated with central nervous system relapse in patients with diffuse large B-cell lymphoma: Results of a multicenter prospective cohort study

**DOI:** 10.18632/oncotarget.12459

**Published:** 2016-10-04

**Authors:** Seok Jin Kim, Jun Sik Hong, Myung Hee Chang, Jeong-A Kim, Jae-Yong Kwak, Jin Seok Kim, Dok Hyun Yoon, Won Sik Lee, Young Rok Do, Hye Jin Kang, Hyeon-Seok Eom, Yong Park, Jong-Ho Won, Yeung-Chul Mun, Hyo Jung Kim, Jung Hye Kwon, Jee Hyun Kong, Sung Yong Oh, Sunah Lee, Sung Hwa Bae, Deok-Hwan Yang, Hyun Jung Jun, Yang Soo Kim, Hwan Jung Yun, Soon Il Lee, Min Kyoung Kim, Eun Kyung Park, Won Seog Kim, Cheolwon Suh

**Affiliations:** ^1^ Department of Medicine, Samsung Medical Center, Sungkyunkwan University School of Medicine, Seoul, Korea; ^2^ Department of Internal Medicine, Gachon University Gil Medical Center, Gachon University College of Medicine, Incheon, Korea; ^3^ Department of Hematology-Oncology, National Health Insurance Service Ilsan Hospital, Goyang, Gyeongki-do, Korea; ^4^ Department of Internal Medicine, St. Vincent's Hospital, the Catholic University of Korea, Seoul, Korea; ^5^ Department of Internal Medicine, Chonbuk National University Medical School & Hospital, Jeonju, South Korea; ^6^ Department of Internal Medicine, Yonsei University College of Medicine, Seoul, Korea; ^7^ Department of Oncology, Asan Medical Center, University of Ulsan College of Medicine, Seoul, Korea; ^8^ Department of Internal Medicine, Inje University College of Medicine, Inje University Busan Paik Hospital, Gaegumdong, Busanjingu, Busan, Republic of Korea; ^9^ Division of Hematology-Oncology, Department of Medicine, Dongsan Medical Center, Keimyung University School of Medicine, Daegu, Korea; ^10^ Department of Internal Medicine, Korea Cancer Center Hospital, Korea Institute of Radiological and Medical Sciences, Seoul, Korea; ^11^ Hematology-Oncology Clinic, National Cancer Center, Goyang-city, Korea; ^12^ Department of Internal Medicine, Korea University Anam Hospital, College of Medicine, Seoul, Korea; ^13^ Department of Internal Medicine, Soon Chun Hyang University, Seoul, Korea; ^14^ Department of Internal Medicine, Ewha Womans University, Seoul, Korea; ^15^ Department of Internal Medicine, Hallym University Sacred Heart Hospital, Hallym University College of Medicine, Chucheon, Korea; ^16^ Department of Internal Medicine, Kangdong Sacred Heart Hospital, Seoul, Korea; ^17^ Division of Hematology-Oncology, Department of Medicine, Wonju Severance Christian Hospital, Yonsei University College of Medicine, Wonju, Korea; ^18^ Department of Internal Medicine, Dong-A University Hospital, Busan, Korea; ^19^ Department of Internal Medicine, Daegu Fatima Hospital, Daegu, Korea; ^20^ Department of Internal Medicine, Daegu Catholic University Medical Center, Catholic University of Daegu School of Medicine, Daegu, Korea; ^21^ Department of Hemato-oncology, Chonnam National University Hwasun Hospital, Jeollanamdo, Korea; ^22^ Department of Internal Medicine, Seoul Medical Center, Seoul, Korea; ^23^ Department of Internal Medicine, Kosin University Gospel Hospital, Busan, Korea; ^24^ Department of Hemato-oncology, Chungnam National University Hospital, Daejeon, Korea; ^25^ Department of Internal Medicine, Dankook University College of Medicine, Cheonan, Korea; ^26^ Department of Medicine, Yeungnam University College of Medicine, Daegu, Korea; ^27^ Department of Internal Medicine, Chung Ang University, Seoul, Korea

**Keywords:** diffuse large B-cell lymphoma, central nervous system, prognosis, lactate dehydrogenase

## Abstract

Central nervous system involvement remains a challenging issue in the treatment of patients with diffuse large B-cell lymphoma. We conducted a prospective cohort study with newly diagnosed diffuse large B-cell lymphoma patients receiving rituximab, cyclophosphamide, doxorubicin, vincristine, and prednisone to identify incidence and risk factors for central nervous system involvement. Among 595 patients, 279 patients received pre-treatment central nervous system evaluation, and 14 patients had central nervous system involvement at diagnosis (2.3% out of entire patients and 5.0% out of the 279 patients). For those patients, median follow-up duration was 38.2 months and some of them achieved long-term survival. Out of 581 patients who did not have central nervous system involvement at diagnosis, 26 patients underwent secondary central nervous system relapse with a median follow-up of 35 months, and the median time to central nervous system involvement was 10.4 months (range: 3.4–29.2). Serum lactate dehydrogenase > ×3 upper limit of normal range, the Eastern Cooperative Oncology Group performance status ≥ 2, and involvement of sinonasal tract or testis, were independent risk factors for central nervous system relapse in multivariate analysis. Our study suggests that enhanced stratification of serum lactate dehydrogenase according to the National Comprehensive Cancer Network-International Prognostic Index may contribute to better prediction for central nervous system relapse in patients with diffuse large B-cell lymphoma. This trial was registered at clinicaltrials.gov identifier: 01202448.

## INTRODUCTION

Diffuse large B-cell lymphoma (DLBCL) is the most common subtype of non-Hodgkin lymphoma, and addition of rituximab to cyclophosphamide, doxorubicin, vincristine, and prednisone chemotherapy (R-CHOP) has improved the outcome of patients with DLBCL [1]. Although several reports suggest that incorporation of rituximab decreased the rate of central nervous system (CNS) relapse [2, 3], the use of rituximab has failed to decrease the incidence of CNS involvement in patients with an age-adjusted International Prognostic Index (IPI) ≥ 2 [4], and clinical outcomes of those patients are still poor in the rituximab era [5–8].

Traditionally, the involvement of certain extranodal sites or nodal lesion such as retroperitoneal lymph nodes is known to confer a higher risk of CNS involvement [6, 9–12]. Some parameters that reflect the tumor burden, including serum lactate dehydrogenase (LDH) and the number of involved extranodal sites were associated with the risk of CNS relapse in previous studies [2, 10]. However, a majority of the studies were retrospective analyses and there is heterogeneity of some crucial factors, such as definition of CNS relapse, methods for CNS evaluation, and whether doing CNS prophylaxis or not. Recently, German High-Grade Non-Hodgkin Lymphoma Study Group proposed a risk model for the prediction of CNS relapse among patients with DLBCL treated with rituximab-containing immunochemotherapy [13]. They defined five conventional IPI factors in addition to involvement of kidney or adrenal gland as factors for CNS relapse (CNS-IPI). The CNS-IPI effectively stratified 2,164 DLBCL patients into 3 risk groups according to the rate of CNS relapse at 2 years. The model was successfully validated among 1,597 patients from the British Columbia Cancer Agency [13].

In DLBCL, the National Comprehensive Cancer Network (NCCN)-IPI showed better prognostication compared to existing standard IPI [14]. Therefore, it would be interesting to evaluate whether enhanced stratification of age or serum LDH contributes to better prediction for CNS relapse. In addition, there has been a scarce of large study regarding CNS relapse of DLBCL among Asian population. Thus, we conducted a multicenter prospective cohort study to evaluate the incidence and risk factors for CNS involvement in patients with DLBCL treated with R-CHOP immunochemotherapy.

## RESULTS

### Patients and overall treatment outcomes

From August 2010 to August 2012, 603 patients were enrolled. Eight patients were excluded because four patients had withdrawn their consent and the other four patients did not meet the inclusion criteria: three patients did not have DLBCL after pathology review, and one patient could not receive R-CHOP due to liver cirrhosis. Thus, the data for 595 patients were finally analyzed, and their median age was 60 years (range: 20– 89). Baseline characteristics of patients were summarized in Table 1. The median cycle number of R-CHOP was six (range: 1–8), and the overall response rate was 90% (534/595) including 480 patients (81%) with a complete response (CR) and 54 (9%) with a partial response. The remaining 61 patients included those with stable disease (*n* = 3), disease progression during treatment (*n* = 38), and an undetermined response (*n* = 20). The reason for an undetermined response was that the patient discontinued treatment after the first or second cycle of R-CHOP due to treatment-related toxicity. At the time of analysis, 164 patients experienced disease progression or relapse, and 141 patients died from the disease (*n* = 93) or other causes (*n* = 48). During the median follow-up duration of 35.0 months [95% confidence interval (CI), 34.2–35.8 months], the 3-year progression-free survival (PFS) and overall survival (OS) were 72% (95% CI, 68.8–75.8) and 75.8% (95% CI, 72.3–79.3), respectively.

**Table 1:** Characteristics of patients

		All patients		Without CNS involvement	
		(*n* = 595)		At diagnosis (*n* = 581)	
Characteristic		*n*	%	*n*	%
Sex	Male	340	57	332	57
Age	≤ 40 years	71	12	70	12
	41–60 years	240	40	234	40
	61–75 years	214	36	208	36
	> 75 years	70	12	69	12
Performance status	ECOG ≥ 2	70	12	68	12
Ann Arbor stage	III/IV	301	51	287	49
Serum LDH	Not increased	308	52	303	52
	× 1~3 ULN	246	41	239	41
	> ×3 ULN	41	7	39	7
extranodal sites (No.	≥ 2	207	35	196	33
Standard IPI (score)	Low (0–1)	265	45	262	45
	Low-intermediate (2)	120	20	118	20
	High-intermediate (3)	112	19	108	19
	High (4–5)	98	16	93	16
NCCN-IPI (score)	Low (0–1)	102	17	102	18
	Low-intermediate (2–3)	280	47	276	47
	High-intermediate (4–5)	164	28	155	27
	High (≥ 6)	49	8	48	8
B symptoms	Presence	145	24	141	24
Serum albumin	< 3.5 g/dL	135	23	129	22
Hemoglobin	< 10 g/dL	83	14	81	14
Platelet count	< 100,000/mm^3^	34	6	33	6
Lymphocyte count	< 1,000/mm^3^	141	24	137	24

ECOG, Eastern Cooperative Oncology Group; LDH, Lactate dehydrogenase; IPI, International Prognostic Index; NCCN, National Comprehensive Cancer Network

### CNS involvement

**Figure 1:**
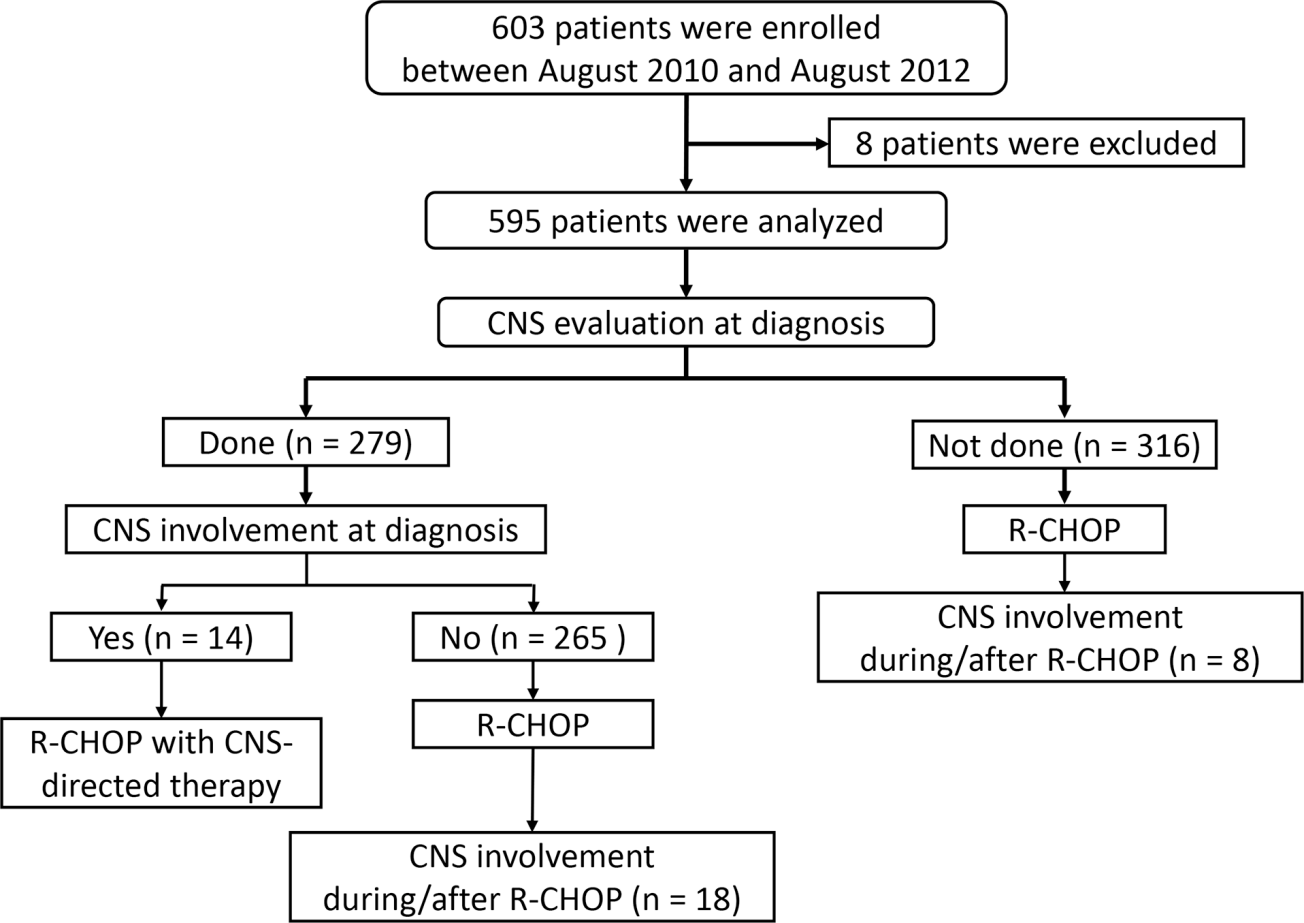
CONSORT diagram of the current PROCESS study

CNS evaluation was performed in 279 patients who had suspicious symptom or sign for CNS involvement or had ≥ 1 risk factors according to the recommendation of the protocol. Fourteen patients had CNS involvement (2.4% out of entire patients and 5.0% out of 279 patients with baseline CNS evaluation; Figure 1). This involvement included the presence of lymphoma cells in the cerebrospinal fluid (CSF) (*n* = 9) and brain parenchymal lesions (*n* = 5). All of them received CNS-directed therapy together with R-CHOP except one patient who died of infection during the first cycle of R-CHOP. During the follow-up, 26 patients underwent CNS relapse. Nineteen of them underwent isolated CNS relapse and 7 patients had synchronous CNS and systemic relapse. The 1-year and 2-year rate of CNS relapse was 3.2% (95% CI, 1.6–4.8) and 4.7% (95% CI, 2.9–6.4%), respectively. Among 265 patients who were initially negative for CNS evaluation, 18 patients (6.9%) had CNS relapse whereas 8 cases (2.5%) of CNS involvement occurred in 316 patients who were not evaluated at diagnosis (Figure 1). Of those 26 patients with CNS relapse, 16 and 24 patients experienced CNS relapse within 1 and 2 years, respectively and the median time to CNS involvement was 10.4 months (range: 3.4–29.2). Among 581 patients excluding 16 patients having CNS involvement at diagnosis, 37 patients received prophylactic intrathecal MTX according to their physicians' decision. Out of these 37 patients, two patients with stage IV disease developed CNS relapse during the follow-up at 11 and 29 months after the diagnosis, respectively.

### Risk factor analyses for CNS relapse

**Figure 2:**
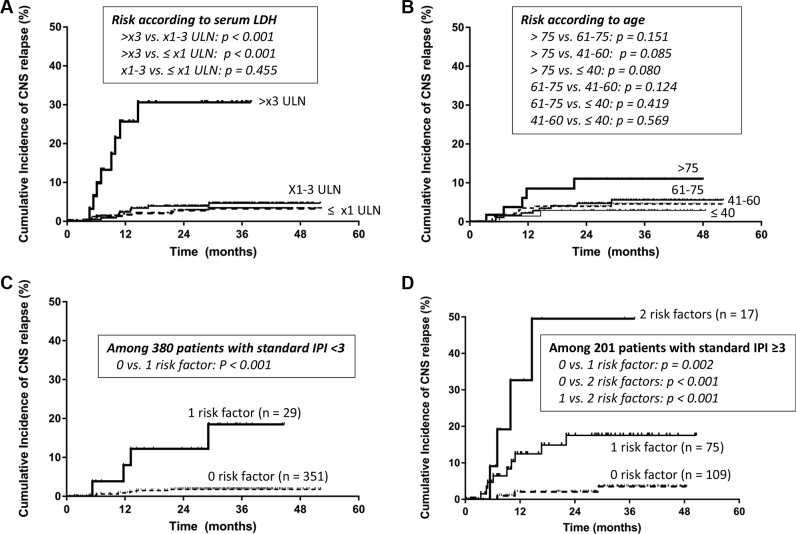
(**A–B**) Cumulative risk of central nervous system relapse according to enhanced stratification of serum lactate dehydrogenase (LDH; A) and age (B); (**C–D**) Cumulative risk of central nervous system relapse according to the number of risk factors from the current study (very high LDH, the Eastern Cooperative Oncology Group performance status ≥ 2, and involvement of sinonasal tract or testis).

Among five IPI factors, Eastern Cooperative Oncology Group performance status (ECOG PS) ≥ 2, involvement of ≥ 2 extranodal sites, and elevation of serum LDH were associated with risk for CNS relapse in univariate analysis, whereas age > 60 (*P* = 0.241) and stage III/IV (*P* = 0.111) were not (Table 2). Patients with very high LDH [> ×3 upper limit of normal range (ULN), according to the NCCN-IPI] were strongly associated with the increased risk for CNS relapse (Figure 2A). However, enhanced analysis according to age failed to show difference, although very old age had a tendency of higher risk of CNS relapse (Figure 2B). Presence of B symptoms, and lower blood cell counts were associated with CNS relapse in univariate analysis (Table 2). Involvement of retroperitoneal lymph node, bone marrow (BM), sinonasal tract, nasopharynx, spleen, and testis were predictive of CNS relapse (Table 3). In contrast, patients with kidney or adrenal gland involvement (35/681= 6.0%; 3 patients had both kidney and adrenal gland involvement) were not associated with risk for CNS relapse. For multivariate analysis, we included LDH > ×3 ULN instead of > ×1 ULN because of its stronger association with CNS relapse. Standard and NCCN-IPI were excluded from the multivariate analysis because they already comprised five individual factors. As a result, very high LDH, ECOG PS ≥ 2, and involvement of testis and sinonasal tract, were independent factors for CNS relapse (Table 4). When we suppose that there are 3 risk factors for CNS relapse (taking testis and sinonasal tract involvement together), patients with more risk factors showed higher cumulative incidence of CNSrelapse, irrespective of higher *vs*. lower risk according to standard IPI (Figure 2C and 2D).

**Table 2:** Pretreatment clinical characteristics and central nervous system involvement

*N* = 581		Total number	CNS event HR	Comparison of actuarial incidence			
				95% CI		*P*	
Sex	Female	249	11	1			
	Male	332	15	1.04	0.48	2.27	0.919
Age	≤ 60 years	304	12	1			
	> 60 years	277	14	1.59	0.73	3.43	0.241
Age (enhanced)	≤ 40 years	70	2	1			
	41–60 years	234	10	1.55	0.34	7.07	0.573
	61–75 years	208	9	1.86	0.40	8.62	0.427
	> 75 years	69	5	3.90	0.75	20.18	0.105
ECOG PS	0/1	513	20	1			
	≥ 2	68	6	3.62	1.45	9.05	0.006
Stage	I/II	294	10	1			
	III/IV	287	16	1.90	0.86	4.19	0.111
Extranodal sites	0/1 vs.	385	11	1			
	≥ 2	196	15	3.21	1.47	6.99	0.003
Serum LDH	Normal	303	9	1			
	Increased	278	17	2.40	1.07	5.39	0.034
serum LDH (enhanced)	≤ ×1 ULN	303	9	1			
	×1–3 ULN	239	9	1.42	0.56	3.58	0.457
	> ×3 ULN	39	8	11.83	4.51	31.10	< 0.001
serum LDH (modified)	≤ ×3 ULN	542	18	1			
	> ×3 ULN	39	8	10.50	4.53	24.31	< 0.001
standard IPI score	< 3	380	10	1			
	≥ 3	201	16	3.90	1.76	8.56	0.001
NCCN-IPI score	< 4	378	14	1			
	≥ 4	203	12	2.14	0.99	4.63	0.054
B symptoms	Absence	440	16	1			
	Presence	141	10	2.25	1.02	4.95	0.045
Serum albumin	> 3.5 g/dL	452	19	1			
	≤ 3.5 g/dL	129	7	1.62	0.68	3.86	0.276
Hemoglobin	> 10 g/dL	500	20	1			
	≤ 10 g/dL	81	6	2.23	0.90	5.56	0.085
Platelet count	> 100,000/mm^3^	548	21	1			
	≤100,000/mm^3^	33	5	5.60	2.10	14.88	0.001
Lymphocyte count	> 1,000/mm^3^	444	16	1			
	≤ 1,000/mm^3^	137	10	2.27	1.03	5.00	0.042

CNS: central nervous system; HR: Hazard ratio; CI: confidence interval; ECOG- Eastern Cooperative Oncology Group; PS: performance status; LDH-Lactate dehydrogenase; IPI: International Prognostic Index

**Table 3:** Relationship of pre-treatment nodal and extranodal involvement to central nervous system relapses among 581 patients

Nodal and extranodal involvement	involvedpatients	CNS event	Comparison of actuarial incidence			
			HR	95% CI		*P*
Node region involvement						
Retroperitoneal	65	6	3.03	1.21	7.56	0.017
Extranodal involvement						
Bone marrow	67	7	3.39	1.43	8.08	0.006
Orbit	7	2	11.79	2.78	50.0	< 0.001
Sinonasal tract	26	6	8.51	3.41	21.24	< 0.001
Nasopharynx	48	6	3.39	1.36	8.45	0.009
Liver	35	3	2.17	0.65	7.23	0.207
Stomach	75	1	3.92	0.53	28.94	0.180
Intestine	89	1	4.56	0.62	33.66	0.137
Bone	64	5	2.23	0.84	5.92	0.107
Spleen	83	7	2.59	1.10	6.17	0.032
Kidney	19	2	3.02	0.71	12.80	0.133
Testis	6	2	8.21	1.94	34.80	0.004
Lung	37	2	1.43	0.34	6.07	0.625
Breast	19	1	1.16	0.16	8.56	0.885
Adrenal gland	19	1	1.55	0.21	11.42	0.669

CNS: central nervous system; HR: Hazard ratio; CI: confidence interval

**Table 4:** Multivariate analysis of the risk of central nervous system involvement

Characteristics	Comparison of actuarial incidence			
	HR	95% CI		*P*
ECOG PS ≥ 2	2.88	1.00	8.27	0.050
Extranodal sites≥ 2	0.93	0.35	2.49	0.892
LDH > ×3 ULN	9.73	3.02	31.41	< 0.001
B symptom	1.23	0.44	3.49	0.694
Hemoglobin ≤10 g/dL	0.41	0.11	1.53	0.183
Platelet ≤100,000/mm^3^	1.97	0.57	6.76	0.283
Lymphocyte ≤1,000/mm^3^	1.42	0.54	3.75	0.483
Retroperitoneal lymph node	1.12	0.34	3.73	0.848
Bone marrow	1.37	0.47	3.99	0.560
Orbit	2.75	0.49	15.35	0.248
Sinonasal tract	12.87	3.57	46.42	< 0.001
Nasopharynx	2.16	0.70	6.66	0.182
Spleen	1.76	0.61	5.04	0.295
Testis	20.52	4.00	105.27	< 0.001

HR: Hazard ratio; CI: confidence interval; ECOG- Eastern Cooperative Oncology Group; PS: performance status; LDH-Lactate dehydrogenase.

### Outcomes of patients with CNS involvement

**Figure 3:**
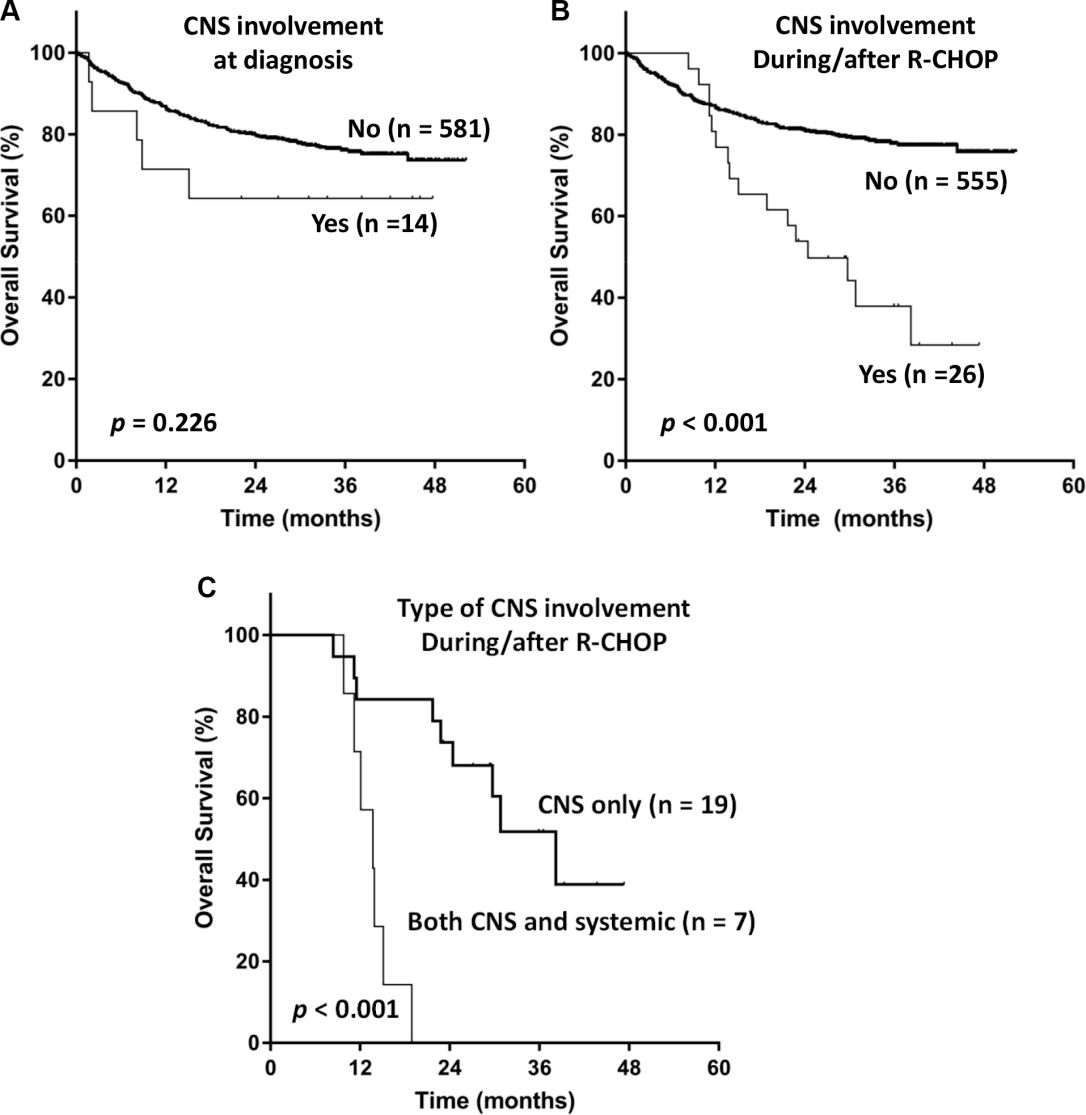
Kaplan-Meier curves for overall survival of patients according to (A) central nervous system involvement (CNS) at diagnosis, (B) CNS involvement during or after immunochemotherapy, and (C) the pattern of CNS involvement during or after immunochemotherapy

Five out of 14 patients who had CNS involvement at diagnosis, died due to systemic disease progression (*n* = 3), infectious complication (*n* = 1), and both (*n* = 1). Median follow-up duration for patients with CNS involvement at diagnosis was 38.2 months (95% CI, 24.8–51.7), and Kaplan-Meier curve for OS showed a plateau (Figure 3A). Among 26 patients with CNS relapse, 16 patients died due to systemic disease progression (*n* = 9) and infectious complication (*n* = 7). The OS of patients with CNS relapse was dismal (Figure 3B). Patients who underwent isolated CNS relapse showed superior OS compared to those who had synchronous CNS and systemic relapse [median OS 38.2 months (95% CI, 26.7–49.6) vs. 13.7 months (95% CI, 9.5–17.9), *P* < 0.001; Figure 3C]. However, a majority of patients with isolated CNS relapse eventually died of progression of CNS or subsequent extra-CNS DLBCL, or infectious complication.

## DISCUSSION

Our study analyzed risk factors for CNS involvement in newly diagnosed DLBCL patients receiving R-CHOP who were registered the prospective cohort. Thus, we prospectively monitored the occurrence of CNS relapse in enrolled patients, and evaluated CNS involvement at diagnosis from patients at risk with CSF study and/or brain imaging. In this study, our patients who had a very high serum LDH, defined as > ×3 ULN, showed a remarkably higher risk for CNS relapse. It was in line with previous studies reporting its association with CNS relapses. Thus, the study analyzing 435 patients with DLBCL from 1999 to 2005 and reporting 7.1% of CNS relapses showed that patients with a LDH > ×1 ULN did not have higher risk for CNS relapse among 309 patients treated with R-CHOP (*P* = 0.267) whereas patients with a LDH > ×2 ULN had a significant association (*P* = 0.026) [3, 15]. These results implied the simple dichotomization of patients according to usual cutoff value of LDH might not be suitable for predicting high risk group of DLBCL because serum LDH could reflect tumor burden and growth. Thus, patients with very high tumor burden as well as rapid growth of tumor cells might have more risk of treatment failure including CNS relapse, and those patients might have very high level of serum LDH. Therefore, higher level of serum LDH might be theoretically correlated with higher risk of CNS relapse because patients with high tumor burden could be more vulnerable to have CNS involvement. In our cohort, sinonasal tract and testis, traditional well-known risk factors for CNS relapse were associated with CNS relapse. On the other hand, the involvement of kidney or adrenal gland was not significantly related with CNS relapse unlike the study reporting CNS-IPI [13]. Similarly, our data showed that not all of five individual IPI factors had a statistically significant association with CNS relapse. Although these discrepancies between our data and that of German group study could be fully explained, it might be related with relatively smaller number of patients in our study (*n* = 581) than that of German group study (*n* = 2,741). Indeed, other variables of CNC-IPI such as age > 60 years and advanced stage showed higher hazard ratio (HR) than those of age ≤ 60 and Ann Arbor stage I/II (HR 1.59 and 1.90, respectively) although they were not statistically significant. Thus, age and stage might become significant factors for CNS involvement if the sample size could be expanded.

Likewise, it was hard to predicate that patients with the involvement of those sites and other sites previously suggested for the site of high risk of CNS relapse were not associated with CNS relapse at all even though the involvement of sinonasal tract and testis were excluded in the CNS-IPI [13]. Extranodal involvement in the craniofacial area has been considered to increase the risk of CNS involvement because this area is anatomically close to skull base, which may allow lymphoma cells to contiguously invade the adjacent leptomeninges and brain [16]. Previous literatures as well as the experience from treating patients with acute lymphoblastic leukemia/lymphoma have been supporting the association of testicular involvement with CNS relapse [6, 10, 12]. Therefore, it would be prudent and reasonable to still suspect that patients with the involvement of those sites may have higher risk for CNS relapse.

The comparison of OS according to CNS involvement showed that the OS of patients was significantly worse than that of patients without CNS involvement. These findings were consistent with the negative impact of CNS relapse on the prognosis of DLBCL patients. However, among 14 patients with CNS involvement at diagnosis, the OS showed a plateau. Although the number was too small and the types of CNS-directed therapy were not uniform, this might be related with the introduction of CNS-directed therapy based on early identification of CNS involvement. Indeed, a previous study supported the usefulness of early intervention of CNS involvement in lymphoma patients because DLBCL patients with CNS involvement at diagnosis maintained their CR after they received MTX (8 g/m^2^) with leucovorin rescue every 2 weeks for 8 cycles, concurrently with R-CHOP every 3 weeks a total of 6 cycles with a median follow-up of 66 months [17]. Therefore, prognosis of patients with synchronous CNS and systemic DLBCL at diagnosis might not be as dismal as those with secondary CNS relapse and a part of patients could achieve long-term survival if they receive adequate CNS-directed therapy.

Our study was a prospective cohort study focusing on the evaluation of CNS involvement including CNS relapse as well as CNS involvement at diagnosis, and it could be discriminated from the majority of studies reporting outcomes and risk factors because they were retrospective studies with relatively small number of patients and heterogeneous treatments and subtypes. Nevertheless, our study had several limitations. First, the diagnosis of leptomeningeal involvement was mainly based on conventional cytology and we did not perform flow cytometry to look for immunophenotypic evidence of CNS involvement. Given flow cytometric analysis would be more accurate for identifying patients with CNS involvement than conventional cytology [18], the use of standardized flow cytometric panels and uniform definitions of positivity might produce different results [19]. Second, we did not make obligation for CNS evaluation at diagnosis, prophylactic intrathecal chemotherapy, and type of CNS-directed therapy for patients with CNS involvement at diagnosis although we provided recommendations to each institute. Therefore, the incidence of CNS involvement at diagnosis might be slightly underestimated, and we could not analyze the role of intrathecal prophylaxis and efficacy of a certain CNS-directed therapy. Finally, we did not analyze the impact of novel morphological or biological classification of DLBCL to the incidence of CNS involvement, including germinal center B-cell (GCB) type DLBCL *vs.* non-GCB type [20], double hit or double protein DLBCL *vs.* not [21], and concordant *vs.* discordant BM involvement of DLBCL [22]. Future studies may define the relationship of the classifications to CNS involvement and provide more effective treatment strategy for patients with CNS involvement of DLBCL.

In conclusion, very high serum LDH was an independent prognostic factor for CNS relapse in addition to ECOG PS ≥ 2 and involvement of sinonasal tract or testis in patients with DLBCL treated with R-CHOP. Future studies on the prognostic role of the enhanced stratification of previously known risk factors and the novel morphological or biological classifications of DLBCL for CNS relapse may contribute to define higher risk group for CNS relapse.

## MATERIALS AND METHODS

### Patients and study design

The inclusion criteria of our PROCESS (Prospective Cohort Study with Risk-Adapted Central Nervous System Evaluation in DLBCL) cohort were patients: (1) with newly diagnosed DLBCL according to the 2008 World Health Organization classification; (2) planned to receive R-CHOP as a primary treatment; (3) with ≥ 20 years of age; (4) with written informed consents. Patients with primary DLBCL of the CNS (PCNSL) were excluded. The diagnosis of CNS involvement was based on the presence of lymphoma cells in CSF cytology or brain parenchymal lesion on imaging studies. Incidence rate of CNS involvement at diagnosis and CNS relapse during or after R-CHOP chemotherapy were estimated, respectively. The actuarial incidence and analyses of CNS relapse was obtained after excluding patients with CNS involvement at diagnosis and then by censoring patients with follow-up loss or death before the occurrence of CNS involvement. The primary endpoint was incidence and risk factors for CNS relapse. The secondary endpoints were incidence of CNS involvement at diagnosis, OS, PFS, and time to CNS involvement. The OS was calculated from the time of diagnosis to the date of last follow-up or death from any cause whereas PFS was from the date of diagnosis to the date of relapse or progression or the last follow-up or death from any cause. The time to CNS involvement was from the date of diagnosis to the date of CNS relapse, and patients who were died from any cause before the CNS relapse occurred were censored. The study was approved by relevant institutional review boards of the participating institutes.

### Study conduct

The study was conducted at 27 hospitals in Korea belonging to the Consortium for Improving Survival of Lymphoma (CISL) [23]. After enrollment, patients received 3-weekly R-CHOP up to 6–8 cycles. The cycle length could be shortened to 3–4 cycles in patients with stage I/II disease. Although CNS evaluation and prophylaxis were allowed based on each physician's discretion, CNS evaluation via lumbar puncture was recommended if a patient had any symptom or sign suspicious for CNS involvement, or if a patient had at least one of the followings: (1) increased serum LDH; (2) ≥ 2 extranodal involvement; (3) serum albumin < 3.5 g/dL; (4) BM involvement; (5) involvement of retroperitoneal lymph nodes; (6) involvement of testis, breast, bone, sinonasal tract, orbit, kidney/adrenal gland, or epidural spaces; or (7) with human immunodeficiency virus infection. Brain magnetic resonance imaging and/or CSF re-examination were recommended in case of abnormal results in CSF analysis such as pleocytosis despite negative cytology. For patients with CNS involvement at diagnosis, CNS-directed therapy such as intrathecal or intravenous methotrexate (MTX) was added to R-CHOP based on each institute's policy. Intrathecal prophylaxis was allowed for those without CNS involvement at diagnosis but seem to be at risk of CNS relapse, based on each physician's discretion. Once the occurrence of CNS involvement was found, it was recorded and regularly reported to the central office of the CISL. The follow-up data including survival and disease status were updated and centrally reviewed every 6 months.

### Statistical analysis

Fisher's exact test was applied to identify associations between categorical variables. The time variable was estimated based on Kaplan-Meier curves and compared using the log-rank test. Following characteristics were evaluated as a potential risk factor for CNS relapse based on review of literature: five standard IPI factors [5, 7, 24], B symptom [7], involvement of retroperitoneal lymph node [11], kidney or adrenal gland [3], breast [9, 10], and testis [6, 10, 12]. Several extranodal sites of head or upper neck close to brain [orbit, sinonasal tract (nasal cavity and paranasal sinus), and nasopharynx], and sites of known poor prognostic implication (liver, spleen, bone, intestine, and lung) [14] were also evaluated. Serum albumin [11], anemia, thrombocytopenia, and lymphopenia were analyzed. Cox-regression hazard model was used for univariate and multivariate analysis. Variables with a *P* < 0.1 in univariate analysis were included into the multivariate analysis. Two-sided *P* values < 0.05 were considered statistically significant using SPSS version 18.0 (Statistical Package for the Social Sciences Inc., Chicago, IL, U.S.A.).
